# How physical multimorbidity affected the mental health and healthcare utilization of patients with severe mental illness during the COVID-19 pandemic and earthquakes

**DOI:** 10.3389/fpsyt.2025.1590037

**Published:** 2025-06-20

**Authors:** Sarah Levaj, Ivona Šimunović Filipčić, Sara Medved, Laura Shields-Zeeman, Felix Bolinski, Filip Luka Mikulić, Martina Rojnić Kuzman

**Affiliations:** ^1^ Department of Psychiatry and Psychological Medicine, University Hospital Centre Zagreb, Zagreb, Croatia; ^2^ Faculty of Dental Medicine and Health, Josip Juraj Strossmayer University of Osijek, Osijek, Croatia; ^3^ Netherlands Institute of Mental Health and Addiction (Trimbos Institute), Utrecht, Netherlands; ^4^ Emergency Medicine Department, Krapina-Zagorje County, Krapina, Croatia; ^5^ School of Medicine, University of Zagreb, Zagreb, Croatia

**Keywords:** comorbidity, multimorbidity, severe mental illness, COVID-19 pandemics, earthquake, double disaster, health services

## Abstract

**Background:**

People with severe mental illness (SMI) commonly have chronic physical illnesses (CPI) and multimorbidity (CPM). The study aimed to investigate the association between mental health, healthcare utilization, and physical comorbidities in SMI patients affected by the double disasters in Croatia (two earthquakes and the COVID-19 pandemic), and identify predictors of CPI and CPM in this population.

**Method:**

A prospective study was conducted among 90 SMI patients at two-time points: May/June, 2020 (first COVID-19 wave and earthquake) and December, 2020/January, 2021 (second COVID-19 wave and earthquake).

**Results:**

At the first study point, the CPM group showed significantly higher severity of insomnia (p=.003; mean ISI scores (SD): 5.60(4.360), 9.47(6.802), and 10.83(8.026) for no-comorbidity group, CPI and CPM respectively), while CPI group showed higher perceived stress levels (p=.026; mean PSS scores (SD): 18.21(6.882), 22.32(4.619) and 21.67(3.343) for no-comorbidity group, CPI and CPM respectively) compared to the no-comorbidity group. CPI (5/18) and CPM (10/23) groups visited other specialized non-mental health care services significantly more frequently than those without comorbidity (7/46) (χ2 = 6.557, p=.038). A lower score of perceived social support by friends predicted CPI (OR=0.549; 95% CI:0.349-0.864; p=.010, corrected p=.04), but a higher score on alexithymia subscale “difficulty identifying feelings” predicted CPM (OR=1.235; 95% CI:1.071-1.424; p=.002, corrected p=.004).

**Conclusion:**

People with SMI and CPI are especially vulnerable during the COVID-19 pandemic and disasters, as it affects their mental and physical health, leading to increased healthcare utilization. An integrated approach to treating SMI and CPI is necessary for the emergency health response.

## 1 Introduction

Over the last several decades, researchers have consistently reported the high prevalence of chronic physical illnesses (CPI) and multimorbidity (CPM) (≥2 CPI) in individuals diagnosed with severe mental illness (SMI) (1–5). Previous studies have discovered that individuals with SMI have a life expectancy of 20 years less than those without, primarily due to co-existing CPI and limited access to physical healthcare (4, 5).

Multiple factors have been suggested to explain multimorbidity in patients with SMI, including the duration of SMI, poor response to treatment, the presence of negative and cognitive symptoms that may lead to a sedentary lifestyle and unhealthy eating habits, high smoking rates, and medication side effects (1, 4–7). However, individuals experiencing first-episode psychosis exhibit elevated levels of CPI/CPM, even prior to the onset of symptoms (1, 8, 9), which may indicate a shared vulnerability to stressors. Indeed, altered stress response which is common for many (psycho) somatic disorders (10, 11), was also found among patients with psychotic disorders, indicated by higher perceived levels of distress, alterations of cortisol levels, and subsequent altered immune response in response to daily hassles, and this was the case even after treatment (12, 13). An impaired stress response in SMI and CPI/CPM may result from different factors (14), including early trauma (4, 10, 11, 15–17), the inability to recognize emotional states or to cope with negative emotions (18, 19) and alexithymia (20–22). As SMI patients often have lowered or disturbed emotional perception, emotional expression, and alexithymia (23, 24), they may more vulnerable to stress, especially if they have co-morbid somatic disorders (25).

During the COVID-19 pandemic, it has been found that individuals with SMI had a higher incidence of coronavirus disease (COVID-19), a higher risk of developing complicated forms of COVID-19, and higher mortality rates from COVID-19 compared to the general population largely attributed to the presence of comorbidities (26–28). At the same time, psychiatric services across the globe were reduced to emergency care only in many countries during the COVID-19 pandemic, leading to a significant reduction in access to care for those in need of healthcare services worldwide (29). In parallel, the findings from the study examining the direct effects of the COVID-19 pandemic on the delivery of general inpatient care in Croatia, demonstrated a 21% reduction in overall hospital admissions across the hospital network during the pandemic in 2020 (30), reflecting a broader decline in healthcare utilization. This substantial reduction in hospital admissions appears to have been driven by a combination of factors, including enforced lockdowns and quarantines, systemic reorganization of hospital operations, constraints in the availability of the medical workforce, and increased public hesitancy to seek inpatient care (30). While the pandemic spread, Croatia experienced two major earthquakes on March 22^nd^ and December 29^th,^ 2020, leading to additional challenges for both mental health service users and care providers (31, 32).

In this study, we first aimed to examine the associations between CPI and CPM, the severity of mental health problems and the use of health services by persons with SMI in double disasters (pandemic and earthquakes). We hypothesized that a higher number of physical comorbidities in persons with SMI was associated with increased severity of mental health problems and higher use of health services during the COVID-19 pandemic and earthquakes. Secondly, we aimed to identify predictors of CPI and CPM (defined as ≥2CPI) among persons with SMI.

## 2 Materials and methods

### 2.1 Study overview

The study was conducted at the Department of Psychiatry and Psychological Medicine, University Hospital Centre (UHC) Zagreb from May, 2020 until January, 2021 as part of a larger hybrid effectiveness-implementation trial (the RECOVER-E project (LaRge-scalE implementation of COmmunity based mental health care for people with seVere and Enduring mental ill health in EuRopE); see KBC ZAGREB (33–35). The study reported herein entails a prospective survey administered at two-time points during the COVID-19 pandemic. The first measurement was conducted during the first pandemic wave (April-July, 2020) (36) and following the Zagreb earthquake. The second measurement was conducted during the second pandemic wave (September, 2020–February, 2021) (36) and following the Petrinja (central Croatia) earthquake (December 29th, 2020). The detailed circumstances of the study are described elsewhere (32).

### 2.2 Participants

Participants with SMI (schizophrenia and other psychotic disorders (F20-F29), bipolar disorder (F31), or major depressive disorder (F32, F33); according to ICD-10 (International Classification of Diseases 10th Revision)), were recruited consecutively at UHC Zagreb from December, 2018 if they met the eligibility criteria, agreed to participate, and gave informed consent. Other inclusion and exclusion criteria are described elsewhere (32, 34). Data on participants were used in accordance with the regulations on personal data in clinical research according to Resolution No. 52 of July 24^th,^ 2008. OJ n.190 of August 14^th,^ 2008, and in accordance with the regulations of General Data Protection Regulation EU, 2016/679, i.e., each participant was assigned a three-digit number that represents an identification mark on all questionnaires, and all data are marked with the assigned code.

All participants previously involved in the RECOVER-E project were offered to participate in this additional research on the impact of the COVID-19 pandemic and the earthquake on mental health. The Ethics Committee of UHC Zagreb approved the project (July 18^th,^ 2018, Class: 8.1-18/149-2, Number: 02/21 AG), as well as this extension of the research related to the COVID-19 pandemic and the earthquake (May 4^th,^ 2020).

### 2.3 Tools and measures

Patients were interviewed by researchers via telephone-administered surveys at two-time points: (i) from May to June, 2020 and (ii) from December, 2020 to January, 2021.

#### 2.3.1 Socio-demographic data

Socio-demographic data included age, sex, marital status, education, current employment, household size, and structure.

#### 2.3.2 Medical data and utilization of services

Medical data was obtained from available medical records, and by interview and it contained information about whether a participant had been in quarantine or contracted COVID-19 at the moment of the survey, the leading psychiatric diagnosis, prescribed medications, and the presence of co-morbid chronic physical illness (CPI and CPM). CPI was defined as a non-mental health condition requiring medical treatment. In line with our previous studies, chronicity was operationalized as a duration of illness or ongoing somatic therapy of at least six months (6, 37). CPM was defined as having two or more CPIs. Utilization of available medical services (emergency room (ER), general practitioner (GP), psychiatrist, and other specialties) during the COVID-19 pandemic was reported by the participants.

#### 2.3.3 Severity of mental health problems

We defined mental health problems among patients with SMI by the presence of symptoms of insomnia, depression, anxiety, severe stress, and alexithymia.

- Insomnia severity was assessed using the Insomnia Severity Index (ISI), a seven-item questionnaire that assesses the quality of sleep in the past two weeks. A 5-point Likert scale (0–4) is used to rate each item, according to the severity of the problem. The total score ranges from 0 to 28, and higher scores indicate greater sleep difficulties (38).
- The Depression Anxiety Stress Scale-21 (DASS-21) was used for assessing symptoms of depression, anxiety, and stress (39). DASS-21 has 21 items rated from 0 (did not apply to me at all) to 3 (applied to me almost completely or most of the time).
- The Perceived Stress Scale (PSS-10) is a 10-item questionnaire originally, and it is the most widely used psychological instrument for measuring the perception of stress. It consists of 10 items, using Likert’s s Scale for scoring. The total score ranges from 0 to 40, with higher scores indicating higher perceived stress (40).
- The Toronto Alexithymia Scale (TAS-20) is the most commonly used measure of the alexithymia construct (41). It is a 20-item scale; each item was rated on a five-point Likert-type scale, ranging from “strongly disagree” (scored 1) to “strongly agree” (scored 5), with scores ranging from 20 to 100. Higher total scores indicated more alexithymia. TAS-20 has three subscales that assess difficulty identifying feelings (DIF), difficulty describing feelings (DDF) to others, and externally oriented thinking (EOT).

Listed questionnaires regarding mental health problems were used in this study at two-time points, except BRCS and TAS-20, which were used only once.

#### 2.3.4 Coping mechanisms

Coping mechanisms were assessed at first assessment using Brief Resilient Coping Scale (BRCS). Participants circle the answers on a five-point scale (from 1 = strongly disagree to 5 = strongly agree) and the total sum score ranges from 4 to 20. A higher score indicates high resilient coping, and a lower one indicates low resilient coping (42).

#### 2.3.5 Perceived social support

Perceived social support was assessed using The Multidimensional Scale of Perceived Social Support (MSPSS), is a 12-item scale designed to measure perceived social support from three sources: family, friends, and a significant other (43). There are four items for each source of social support. On a scale from 1 (I do not agree at all) to 7 (I completely agree), the participant indicates the degree that best suits real life. Subscales (support from family, friends, and others) are scored separately. Only the results from the first study point were considered.

### 2.4 Statistical analysis

Descriptive analysis was used for sample description. For the primary outcome analysis (the associations of the number of comorbidities (none, CPI, and CPM) and the severity of mental health problems), ANOVA was used to assess the differences in mental health problems between the three clinical groups (CPI, CMP, and no-comorbidity groups) in the two study points. For the secondary outcome analysis (the associations of the number of comorbidities (none, CPI, and CPM) and the utilization of mental health services), Pearson χ2 was used to assess the difference in health care services utilization among the three clinical groups (CPI, CMP, and no-comorbidity groups). For the exploratory outcome analysis, multinomial regression was used to predict the presence of somatic comorbidities (CPI and CPM) from age, sex, level of education, living conditions, alexithymia and stress coping mechanisms. The results were interpreted at the 5% significance level (α = 0.05). The statistical program STATA/IC 15.1 Stata Corp LLC was used for statistical analysis (see STATA/IC). To correct for multiple testing, we applied Holm-Bonferroni sequential correction (44) using the Excel calculator (45).

## 3 Results

We enrolled a sample of 90 outpatients diagnosed with SMI. Sociodemographic and medical data at baseline are presented in **Table 1**. Within the sample, we found that CPI were present in 42/90 (46.7%) of patients, CPM in 23/90 (25.6%) and 48/90 (53.3%) without physical comorbidity. The most prevalent CPI were diseases of the musculoskeletal system and connective tissue, endocrine, nutritional and metabolic diseases, and autoimmune diseases.

**Table 1:** Participants’ sociodemographic and medical data.

Variable	N	(%)
Socio-demographic characteristics		
Gender, men	39	(43.3)
Age (years), mean (SD)*	41.9	(14.6)*
Finished high school	79	(90.8)
Single/divorced	62	(68.9)
Living alone	12	(13.3)
Mean number of persons in household (SD) *	2.7	(1.3)*
Mean number of children in household (SD)*	0.3	(0.7)*
Work status		
employed	20	(22.7)
unemployed	42	(47.7)
retired	21	(23.9)
Psychiatric diagnosis		
Schizophrenia and other psychotic disorders	63	(70.0)
Major depressive disorder	19	(21.1)
Bipolar-affective disorder	8	(8.9)
Psychiatric Medication		
Oral antipsychotics	79	(88.8)
Long acting injectable antipsychotics (LAIs)	23	(27.4)
Mood stabilizers	26	(29.2)
Antidepressants	34	(38.2)
Sedatives	56	(62.9)
Number of Chronic physical illness, mean (SD)		
None	48	(53.3)
CPI (one chronic physical illness)	19	(21.1)
CPM (≥2CPI)	23	(25.6)
Chronic physical illnesses		
Diseases of the circulatory system	5	(5.6)
Endocrine, nutritional and metabolic diseases	9	(10.1)
Autoimmune diseases	7	(7.9)
Neoplasms	3	(3.4)
Diseases of the musculoskeletal system and connective tissue	10	(11.2)
Diseases of the blood and blood-forming organs and certain disorders involving the immune mechanism	2	(2.2)
Diseases of the nervous system	5	(5,6)
Diseases of the digestive system	1	(1,1)

Data are presented as number (percentage) of participants if not stated otherwise.

(N =90) * unless otherwise specified. SD, standard deviation; CPI, Chronic physical illness; CPM, chronic physical multimorbidity.

### 3.1 Associations of CPI and CPM with mental health problems

In the first time point, the three clinical groups (CPI, CMP, and no-comorbidity groups) differed in insomnia severity (mean ISI scores (SD): 5.60 (4.360), 9.47(6.802) and 10.83 (8.026) for no-comorbidity group, CPI and CPM respectively, p=.002, corrected 0.004) with *post-hoc* test showing significantly higher insomnia severity in patients with CPM (p=.003) compared to patients with no-comorbidity. Stress levels were also different between the groups (mean PSS scores (SD): 18.21 (6.882), 22.32 (4.619) and 21.67 (3.343) for no-comorbidity group, CPI and CPM respectively, p=.01, corrected p=.01), with CPI (p=.026) patients showing significantly higher levels of stress compared to no-comorbidity patients. In the second assessment, these differences were not significant anymore (**Table 2**). This was mainly due to the levels of the severity of insomnia and perceived stress rising among patients with no-comorbidities as well, close to the levels of the two other groups.

**Table 2:** Differences in mental health problems between the three clinical groups (no comorbidity, CPI, CPM) in the two study points.

	1st assessment				2nd assessment		
		N	M (SD)	F (p)	N	M (SD)	F(p)
ISI	None	48	5.60 (4.360)	6.851 (**0.002**)	44	8.52 (5.853)	1.670 (0.195)
	CPI	19	9.47 (6.802)		17	10.76 (6.505)	
	CPM	23	10.83 (8.026)		22	11.14 (6.394)	
DASS-21	None	48	15.26 (16.056)	2.234 (0.113)	44	14.93 (13.414)	2.111 (0.128)
	CPI	19	23.42 (13.570)		17	23.59 (16.264)	
	CPM	23	21.65 (18.225)		22	18.95 (17.236)	
PSS	None	48	18.21 (6.882)	4.879 (**0.010**)	45	19.73 (5.479)	1.780 (0.128)
	CPI	19	22.32 (4.619)		17	22.47 (3.907)	
	CPM	23	21.67 (3.343)		22	20.82 (5.252)	

M, arithmetic mean; SD, standard deviation; F, F-statistic (from ANOVA); Significant p values are shown in bold; CPI, chronic physical illness; CPM, chronic physical multimorbidity; ISI – Insomnia Severity Index; DASS-21, The Depression Anxiety Stress Scale-21; PSS, Perceived Stress Scale.

### 3.2 Utilization of medical services according to number of comorbidities

Data on utilization of medical services are presented in **Table 3**. There was no significant difference between the three groups (patients with no comorbidities, CPI and CPM) in the first assessment, except for the frequency of visits to specialized care doctors other than mental healthcare, where patients with CPI (5 out of 18) and CPM (10 out of 23) visited other specialized care doctors more frequently than those with no-comorbidity (7 out of 46) (χ2 = 6.557, p=.038). This difference was not found to persist in the second assessment.

**Table 3:** Utilization of medical services during the two study points in patients with SMI according to the number of co-morbidities.

	1^st^ assessment				2^nd^ assessment			
	None	CPI	CPM	χ2 (p)	None	CPI	CPM	χ2 (p)
Emergency setting								
yes no	7 40	2 17	4 19	0.474(.788)	12 33	4 13	4 217	0.458 (.795)
Hospital treatment								
yes no	10 37	2 17	2 20	2.188 (.335)	23 21	11 8	16 7	1.856 (.395)
Family medicine								
yes no	23 21	11 8	16 7	1.856(.395)	36 9	15 2	15 7	2.408 (.300)
Psychiatry service								
yes no	28 18	11 8	14 9	1.846 (.764)	38 6	15 2	19 2	0.228 (.892)
Other specialized care								
yes no	7 39	5 13	10 13	**6.557 (.038)**	15 28	10 6	13 9	5.369 (.068)

CPI, chronic physical illness; CPM, chronic physical multimorbidity; χ2 test = chi-square test; Significant p values are shown in bold.

At the first assessment, none of the participants reported having contracted COVID-19. However, one was self-isolated because of contact with an infected person, whereas at the second assessment, one participant was infected, and three self-isolated.

### 3.3 Predictors of CPI and CPM in persons with SMI

CPI was predicted only by low perceived social support by friends (OR = 0.549; 95% CI 0.349-0.864; p=.010, corrected p=.04), indicating that those who had more perceived social support by friends were less likely to have CPI. CPM was significantly predicted by older age (OR= 1.066; 95% CI 1.007-1.130; p=.029), but also by alexithymia low scores of subscales ‘‘difficulty describing feelings’’ (OR =0.745; 95% CI 0.573-0.969; p=.028), but both significances were lost after correction for multiple testing (both corrected p=.084). However, after correction for multiple testing, CPM was also predicted by higher scores ‘‘difficulty identifying feelings’’ (OR =1.235; 95% CI 1.071-1.424; p=.002, corrected p=.004). Details are shown in **Table 4**.

**Table 4:** Predictors of chronic physical illness (CPI) and chronic physical multimorbidity (CPM) in patients with SMI.

Variable	Chronic physical illness			Chronic physical multimorbidity		
	P	OR	95% CI	P	OR	95% CI
Gender (female)	.071	0.223	0.044-1.137	.980	0.980	0.203-4.729
Age	.228	1.035	0.979-1.094	**.029**	**1.066**	**1.007-1.130**
Education	.139	0.790	0.579-1.079	.590	0.944	0.766-1.164
Living conditions	.136	0.123	0.008-1.937	.335	0.342	0.39-3.022
BRCS	.907	0.987	0.796-1.225	.590	0.944	0.766-1.164
MPSS						
total score	.333	1.276	0.779-2.091	.804	0.949	0.625-1.439
friends	**.010**	**0.549**	**0.349-0. 864**	.058	0.646	0.411-1.015
Others						
Family	.919	0.972	0.563-1.678	.941	0.982	0.604-1.596
TAS-20						
DDF	.358	0.887	0.687-1.146	**.028**	**0.745**	**0.573-0.969**
DIF	.229	1.088	0.948-1.249	**.002**	**1.235**	**1.071-1.424**
EOT	.750	0.977	0.846-1.128	.205	1.096	0.951-1.263

OR, odds ratio; CI, confidence interval; p, statistical significance; Significant p values are shown in bold; BRCS, Brief Resilient Coping Scale; MPSS, The Multidimensional Scale of Perceived Social Support; TAS-20, Toronto Alexithymia Scale; DIF, difficulty identifying feelings; DDF, difficulty describing feelings to others; EOT, externally oriented thinking.

## 4 Discussion

We aimed to analyze the associations of the number of comorbidities in patients with SMI and severity of mental health problems and utilization of health services when experiencing double disasters. As we found that almost 50% of SMI patients in our sample have comorbidities, these results are compatible with available reports showing higher prevalence of physical multimorbidity in patients with schizophrenia spectrum compared to the general population, with some studies reporting rates as high as 60-80% (46). At the first time point, the patients with physical comorbidity showed significantly higher severity of insomnia and perceived stress levels compared to the no-comorbidity group, which is consistent with our hypothesis. Since early reports indicated that COVID-19 occurs in severe forms, especially for people with poor physical health, high cardiovascular risk, and obesity (47), it could have caused more distress in this specific population (48, 49). Interestingly, by the time of the second study point, the differences were lost, likely because the perceived levels of stress and insomnia increased among those without physical comorbidities as well. Concordantly, patients with CPI and CPM visited other specialized (non-mental health care) doctors significantly more frequently than those without physical comorbidities in the same study point. This may reflect the possible deterioration of their somatic conditions during the COVID-19 pandemic (50), which is in line with reports showing that the general population’s health was deteriorating at that time (51, 52). These differences in healthcare utilization were lost over time due to the increased utilization of somatic specialist care among patients without comorbidities. Again, this aligns with the studies in the general study population (53). Broader changes in the healthcare system during the pandemic (30, 54) —particularly those related to access to services as explained in the introduction section—should be considered when interpreting these findings, as they likely influenced participants’ healthcare-seeking behaviors and, consequently, the overall study outcomes.

In our study, CPM in SMI patients was significantly predicted by alexithymia scores, while CPI was predicted by low social support from their friends.

Alexithymia has not been extensively researched in persons with SMI, even though emotion regulation is considered a well-known problem in the management of chronic diseases (55) and may affect immune function, according to some studies (18, 19). We found that CPM was significantly predicted by the alexithymia subscales score of more ‘‘difficulty identifying feelings’’. This is concordant with the results showing that alexithymia may increase proneness to somatic and psychiatric disorders in addition to genetic factors, possibly through the disruptions of homeostasis in autonomic, endocrine, and immune functions and increase proneness to chronic distress (25). Patients with alexithymia have limited ability to adapt to stressful situations and tend to engage in unhealthy behaviors, including poor nutrition, excessive alcohol consumption, reduced physical activity, and sedentary lifestyles (20–22) which may all contribute to the development of illnesses. Indeed, difficulty identifying feelings has also been associated with an increased risk of uncontrolled hypertension and Crohn’s disease, possibly due to impaired emotional regulation and increased physiological reactivity to stress (56, 57). Furthermore, due to their difficulty in identifying feelings, patients with alexithymia may have difficulty maintaining close relationships with others and utilizing social supports appropriately to protect themselves from potentially pathological influences of stressful events (22, 58). Indeed, we found that perceived low social support from friends further increases the risk of developing comorbidities. This is in line with many reports indicating that poor social support can further compromise patients’ mental and physical health (59), possibly by compromising immune systems, increasing inflammation, and increasing their vulnerability to chronic physical illnesses and multimorbidity (60). However, we also found that the two alexithymia subscales indicate associations with CPM in opposite directions. While most researchers agree that ‘difficulty identifying feelings’ and ‘difficulty describing feelings’ are salient features of alexithymia, some have suggested that the ‘externally oriented thinking’ subscale differs significantly from ‘difficulty identifying feelings’ and ‘difficulty describing feelings’ (61, 62), which may explain the absence of the associations of the total alexithymia scores and CPI/CPM in our findings. Given the pandemic’s impact on the prevalence of various psychopathologies, notably the increased rates of anxiety and depression, the identification of high-risk groups helps to properly support these individuals (63, 64).

### 4.1 Limitations

There are several important limitations that should be taken into account. Firstly, the assessments relied on self-reporting and may therefore potentially cause over- or under-reporting. The self-reported measures have limited accuracy compared to an interview conducted by a psychiatrist, introducing a possibility for bias. Thus, the discrepancy could be attributed to inadequate detection or recording of mental health problems and physical illnesses. To reduce this potential bias, we utilized available information from medical documentation to complement self-reported data and enhance the reliability. Secondly, the longer-term effects of the COVID-19 pandemic are not addressed, as some of the effects of the disruption of healthcare services may become evident only after a longer period. The third limitation was our sample’s heterogeneity concerning different diagnoses considered SMIs. The fourth limitation of the study pertains to the inability to ascertain whether the observed differences in physical healthcare utilization among participants were driven by individual-level behavioral factors or by systemic constraints in service availability.

Finally, the sample size of participants was rather small and not powered, since the present study was conceived only after the COVID-19 outbreak and *post hoc* power analyses are discouraged (65), which poses limitations for statistical power and generalizability.

## 5 Conclusion

In this study, we confirmed that the presence of comorbid physical illnesses and multimorbidity are common problems that often coexist in the SMI population. The coexistence of comorbid physical illnesses in individuals with SMI in the context of the COVID-19 pandemic and earthquakes may have further affected their mental health by increasing the perceived level of stress and insomnia, which may have led to chronic distress and worsening of their somatic disorders during the COVID-19 pandemic and earthquakes as reflected by the higher utilization of specialized non-mental health care. This calls for an integrated liaison approach in treating persons with SMI, now in the context of pandemics (29) and emergencies, coupling psychosocial interventions that reduce distress together with somatic care.

## Data Availability

The raw data supporting the conclusions of this article will be made available by the authors, without undue reservation.
